# U shape association between triglyceride glucose index and congestive heart failure in patients with diabetes and prediabetes

**DOI:** 10.1186/s12986-024-00819-7

**Published:** 2024-07-02

**Authors:** Yumeng Shi, Chao Yu

**Affiliations:** 1 Department of Cardiovascular Medicine, the Second Affiliated Hospital, Nanchang of Jiangxi, Jiangxi Medical College, Nanchang University, Nanchang, China; 2 Center for Prevention and Treatment of Cardiovascular Diseases, the Second Affiliated Hospital, Jiangxi Medical College, Nanchang of Jiangxi, Nanchang University, Nanchang, China; 3 Jiangxi Provincial Cardiovascular Disease Clinical Medical Research Center, Nanchang, China; 4 Jiangxi Sub-center of National Clinical Research Center for Cardiovascular Diseases, Nanchang, China

**Keywords:** Triglyceride glucose index, Congestive heart failure, Diabetes, Prediabetes, U shape

## Abstract

**Background:**

While previous population studies have shown that higher triglyceride-glucose (TyG) index values are associated with an increased risk of congestive heart failure (CHF), the relationship between TyG and CHF in patients with abnormal glucose metabolism remains understudied. This study aimed to evaluate the association between TyG and CHF in individuals with diabetes and prediabetes.

**Methods:**

The study population was derived from the National Health and Nutrition Examination Survey (NHANES) spanning from 1999 to 2018. The exposure variable, TyG, was calculated based on triglyceride and fasting blood glucose levels, while the outcome of interest was CHF. A multivariate logistic regression analysis was employed to assess the association between TyG and CHF.

**Results:**

A total of 13,644 patients with diabetes and prediabetes were included in this study. The results from the fitting curve analysis demonstrated a non-linear U-shaped correlation between TyG and CHF. Additionally, linear logistic regression analysis showed that each additional unit of TyG was associated with a non-significant odds ratio (OR) of 1.03 (95%CI: 0.88–1.22, *P* = 0.697) for the prevalence of CHF. A two-piecewise logistic regression model was used to calculate the threshold effect of the TyG. The log likelihood ratio test (*p* < 0.05) indicated that the two-piecewise logistic regression model was superior to the single-line logistic regression model. The TyG tangent point was observed at 8.60, and on the left side of this point, there existed a negative correlation between TyG and CHF (OR: 0.54, 95%CI: 0.36–0.81). Conversely, on the right side of the inflection point, a significant 28% increase in the prevalence of CHF was observed per unit increment in TyG (OR: 1.28, 95%CI: 1.04–1.56).

**Conclusions:**

The findings from this study suggest a U-shaped correlation between TyG and CHF, indicating that both elevated and reduced levels of TyG are associated with an increased prevalence of CHF.

## Introduction

Congestive heart failure (CHF), as the terminal stage of various cardiac diseases, has emerged as a significant public health concern impacting residents’ well-being. In the United States alone, CHF affects six million adults over the age of 20 [1, 2], and its mortality rate is rapidly increasing worldwide, imposing a significant social and economic burden on human society [2]. Therefore, to mitigate the incidence and progression of heart failure, it is imperative to concentrate on high-risk cohorts for CHF, such as patients with diabetes and prediabetes.

Diabetes mellitus (DM) constitutes a primary risk factor for cardiovascular outcomes, encompassing HF [3–9]. The prevalence of heart failure among patients with type 2 diabetes in the United States is estimated to be as high as 22% [10]. Furthermore, research has demonstrated that even individuals with pre-diabetes are at an increased risk for developing HF. The pathophysiological basis of diabetes and pre-diabetes is intricately linked to the dysregulation of glucose metabolism and insulin resistance (IR), thus underscoring the paramount importance of identifying pertinent biomarkers. The triglyceride-glucose (TyG) index is widely employed as a surrogate marker for IR in population-based studies due to its ease of acquisition and has been substantiated by numerous investigations [11–13]. Previous studies have established a direct correlation between elevated TyG and structural as well as functional impairment of the left ventricle (LV), accompanied by myocardial fibrosis, consequently increasing the risk of HF [14–17]. However, the aforementioned research has primarily focused on the general population or patients with coronary heart disease, Even in diabetic patients, only a positive correlation between TyG and HF is found, and most diabetic patients are also complicated with coronary heart disease, cardiac insufficiency and other cardiovascular diseases [18–20]. In fact, patients with impaired glucose metabolism and uncomplicated diabetes mellitus represent high-risk populations for heart failure, necessitating increased attention and research efforts. Simultaneously, monitoring TyG levels can serve as a valuable tool to observe disease progression in this cohort, enabling early intervention strategies and improved prognostic outcomes.

To address the research gap concerning the interplay between metabolic factors and heart failure, we leveraged the comprehensive National Health and Nutrition Examination Survey Database, which encompasses a substantial sample size within the US. Our study aimed to investigate the association between the TyG index and CHF among patients with abnormal glucose metabolism.

## Methods

### Study design and population

The data for this cross-sectional survey was obtained from the ongoing National Health and Nutrition Examination Survey (NHANES) project conducted by the National Center for Health Statistics in the United States. This survey aims to assess the nutritional status and health of the general population in a complex, multi-stage sampling design that combines face-to-face interviews surveys with physical examinations [21]. The research plan of NHANES was approved by the Ethics Review Committee of the National Center for Health Statistics. Since 1999, NHANES has evolved into a continuous cross-sectional survey, wherein nationally representative data from continuous NHANES are collected and published biennially. All participants who participated in the research project provided informed consent by signing a document.

**Fig. 1:**
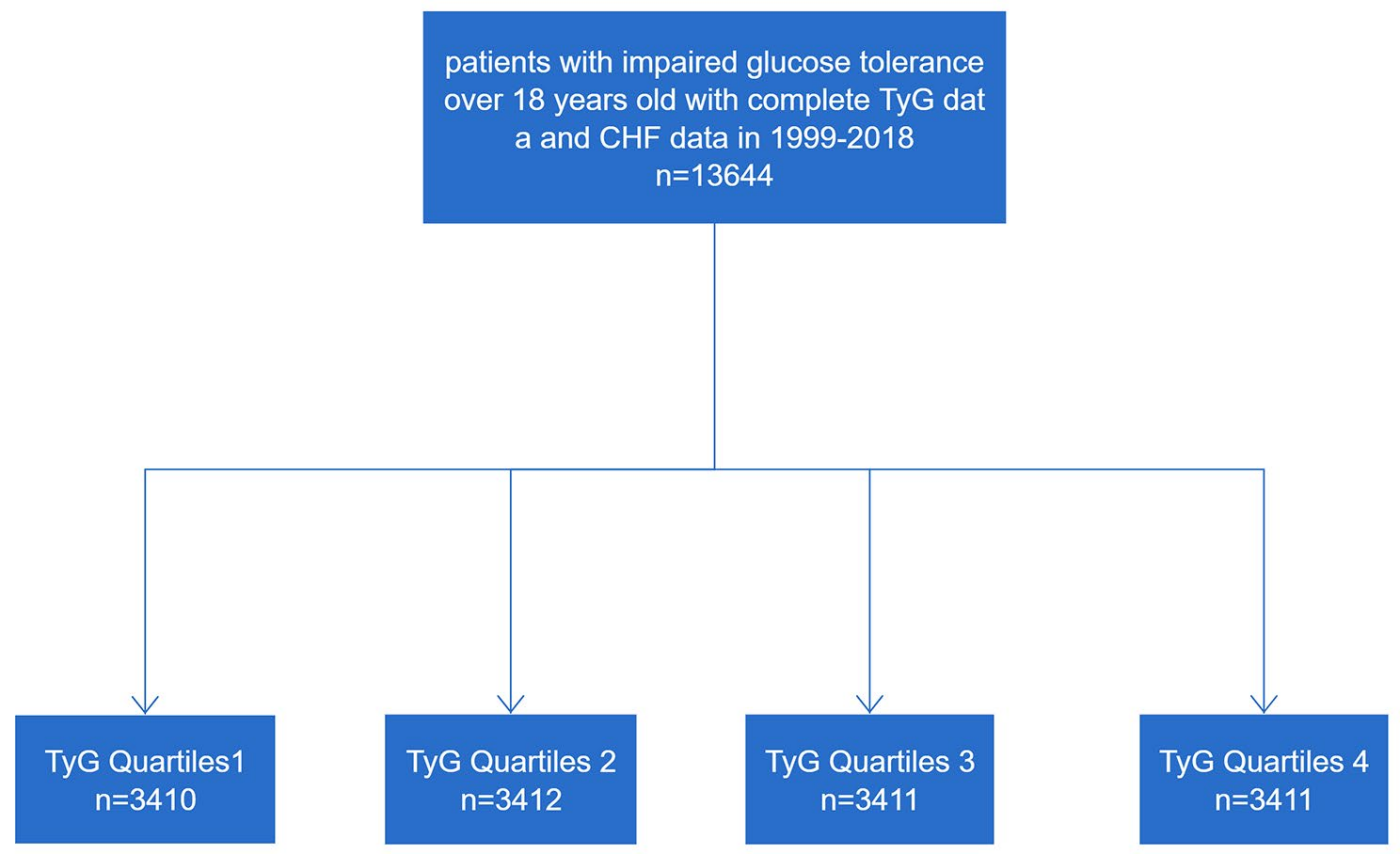
The flow of chart

A total of 13,644 individuals were included in the cross-sectional dataset utilized for this analysis. The inclusion criteria encompassed: (1) Participants aged over 18 years between 1999 and 2018; (2) Availability of complete triglyceride and fasting blood glucose data; (3) Presence of comprehensive questionnaire data on CHF; (4) All participants exhibited impaired glucose tolerance or diabetes (Fig. 1).

### Definition of the TyG and CHF

The exposure variable, TyG, is derived from the combination of triglyceride and fasting blood glucose levels. The specific details are outlined as follows: TyG = ln [TG (mg/dL)*fasting blood glucose (mg/dL)/2]. The outcome variable, congestive heart failure (CHF), is assessed using a face-to-face questionnaire administered by a trained healthcare professional. Specifically, participants who responded affirmatively to the question “Have doctors or other health professionals ever diagnosed you with CHF?” were considered as having chronic heart failure [22].The final event has received extensive validation in previous scholarly literature [23, 24].

### Potential covariates

The covariates considered in this cross-sectional analysis encompass continuous variables, including age, body mass index (BMI), poverty income ratio, glycosylated hemoglobin (HBA1C), estimated glomerular filtration rate (eGFR). Additionally, classified variables such as sex, race, smoking status are also taken into account along with hypertension and hyperlipidemia conditions. Furthermore, the usage of antihypertensive drugs and lipid-lowering drugs is included. The eGFR is calculated using the CKD-EPI formula among the variables considered [25]. BMI can be obtained by dividing weight (kg) by the square of height (m). Prediabetes was defined according to the 2013 American Diabetes Association guidelines as meeting any one of the following criteria: HbA1c between 5.7% and 6.4%, fasting plasma glucose (FPG) between 5.6 mmol/L and 6.9 mmol/L, or a 2-hour plasma glucose value between 7.8 mmol/L and 11.0 mmol/L during an oral glucose tolerance test (OGTT) [26]. Diabetes was defined as a self-reported physician diagnosis of diabetes or having an HbA1c level ≥ 6.5%, FPG level ≥ 7.0 mmol/L, or a 2-hour OGTT plasma glucose level ≥ 11.1 mmol/L.

### Statistical analysis

Continuous variables are represented by the mean ± standard deviation (SD), while categorical variables are presented using frequency and percentage. Following quartile of baseline TyG for describing demographic characteristics, we employed chi-square tests to compare differences among categorical variables and conducted variance (ANOVA) analysis for comparing continuous variables. Logistic regression analysis was employed to calculate the odds ratios (ORs) and 95% confidence interval (CI), enabling evaluation of the independent relationship between TyG and CHF. To this end, we have constructed two models.Model 1 was adjusted for age, sex, race, poverty income ratio, BMI, HbA1c. Model 2 was adjusted for age, sex, race, poverty income ratio, BMI, current smoking, HbA1c, eGFR, serum albumin, hypertension, Hyperlipidemia, antihypertensive drugs, lipoprotein-lowering drugs. Additionally, a generalized additive model and the penalty spline method for fitting smooth curves were used to explore the dose-response relationship between the above-mentioned variables. In the event of detecting nonlinearity, we initially employ a recursive algorithm to compute the inflection point and subsequently construct two two-piecewise logistic models on either side of this inflection point. As an additional exploratory analysis, we conducted subgroup analyses and interaction tests to investigate potential effect modifiers of TyG’s impact on the prevalence of CHF. These subgroups included sex (male vs. female), age(< 65 vs. ≥65), BMI (< 25 vs. ≥25), poverty income ratio (< 2.48 vs. ≥2.48), smoking status (never vs. former vs. now), hypertension (no vs. yes), and hyperlipidemia (no vs.yes).

The significance tests were conducted using bilateral approaches, with a predetermined significance level of 0.05. Empower (R; www.empowerstats.com; X&Y Solutions, Inc., Boston, MA, USA) and the R software package (http://www.R-project.org, The R Foundation) were employed for statistical analysis.

## Results

### Study participants and baseline characteristics

A total of 13,644 participants with impaired glucose tolerance were included in this cross-sectional analysis. The mean age (standard deviation: SD) was 55.83 (16.55) years old, and the average TyG index level (SD) was 8.86 (0.69). Among them, CHF accounted for 4.81% (656 individuals), prediabetes accounted for 6.98% (9,139 individuals), and diabetes accounted for 33.02% (4,505 individuals).

Furthermore, based on the baseline description of the research population in the fourth TyG group, we observed that compared to individuals in the lowest TyG group, those in the highest TyG group exhibited a higher age and a greater proportion of non-hispanic white males and current smokers. Additionally, they demonstrated higher levels of BMI and HBA1C, along with a higher prevalence of hypertension and hyperlipidemia. Moreover, this group displayed increased utilization rates of antihypertensive drugs and lipid-lowering drugs; however, they had lower poverty-income ratios and eGFR levels (Table 1).

**Table 1:** The demographic and clinical characteristics of the population by quartile of baseline TyG

Variable^a^	TyG Quartile				*P* value
	Quartile 1 (< 8.40)	Quartile 2 (8.40to < 8.81)	Quartile 3 (8.81 to < 9.25)	Quartile 4 (≥ 9.25)	
Participants	3410	3412	3411	3411	
Males, *N*(%)	1716 (50.32%)	1833 (53.72%)	1768 (51.83%)	1954 (57.29%)	< 0.001
Age, year	53.19 ± 17.65	55.98 ± 17.04	57.31 ± 15.96	56.85 ± 15.15	< 0.001
BMI, kg/m^2^	28.66 ± 7.25	29.76 ± 7.18	31.07 ± 6.79	31.59 ± 6.51	< 0.001
Race					< 0.001
Non-Hispanic White, *N*(%)	1206 (35.37%)	1455 (42.64%)	1592 (46.67%)	1536 (45.03%)	
Non-Hispanic Black, *N*(%)	1172 (34.37%)	725 (21.25%)	510 (14.95%)	403 (11.81%)	
Mexican American, *N*(%)	396 (11.61%)	584 (17.12%)	661 (19.38%)	845 (24.77%)	
Other Hispanic, *N*(%)	267 (7.83%)	315 (9.23%)	339 (9.94%)	330 (9.67%)	
Other races, *N*(%)	369 (10.82%)	333 (9.76%)	309 (9.06%)	297 (8.71%)	
Current smoking, *N*(%)	598 (17.55%)	695 (20.41%)	678 (19.88%)	729 (21.38%)	< 0.001
Poverty income ratio	2.61 ± 1.62	2.49 ± 1.61	2.46 ± 1.57	2.35 ± 1.57	< 0.001
serum albumin, g/L	41.72 ± 3.48	41.82 ± 3.32	41.82 ± 3.32	41.86 ± 3.49	0.337
HBA1C%	5.69 ± 0.61	5.81 ± 0.73	6.00 ± 0.94	6.89 ± 1.96	< 0.001
eGFR, mL/min/1.73 m^2^	92.86 ± 23.95	88.75 ± 23.55	86.73 ± 23.24	87.22 ± 24.17	< 0.001
hypertension	1580 (46.33%)	1758 (51.54%)	1947 (57.11%)	2116 (62.03%)	< 0.001
Hyperlipidemia	1983 (58.15%)	2591 (75.94%)	3100 (90.88%)	3352 (98.27%)	< 0.001
Antihypertensive drugs	1234 (36.19%)	1356 (39.74%)	1622 (47.55%)	1708 (50.07%)	< 0.001
Lipoprotein-lowering drugs	731 (21.44%)	842 (24.68%)	1003 (29.40%)	1080 (31.66%)	< 0.001

^a^Data are presented as number (%) or mean ± standard deviation

Abbreviation: TyG, triglyceride glucose index; BMI, body mass index; HBA1C: glycosylated hemoglobin, eGFR, estimated glomerular filtration rate

### Associations between TyG and CHF

**Fig. 2:**
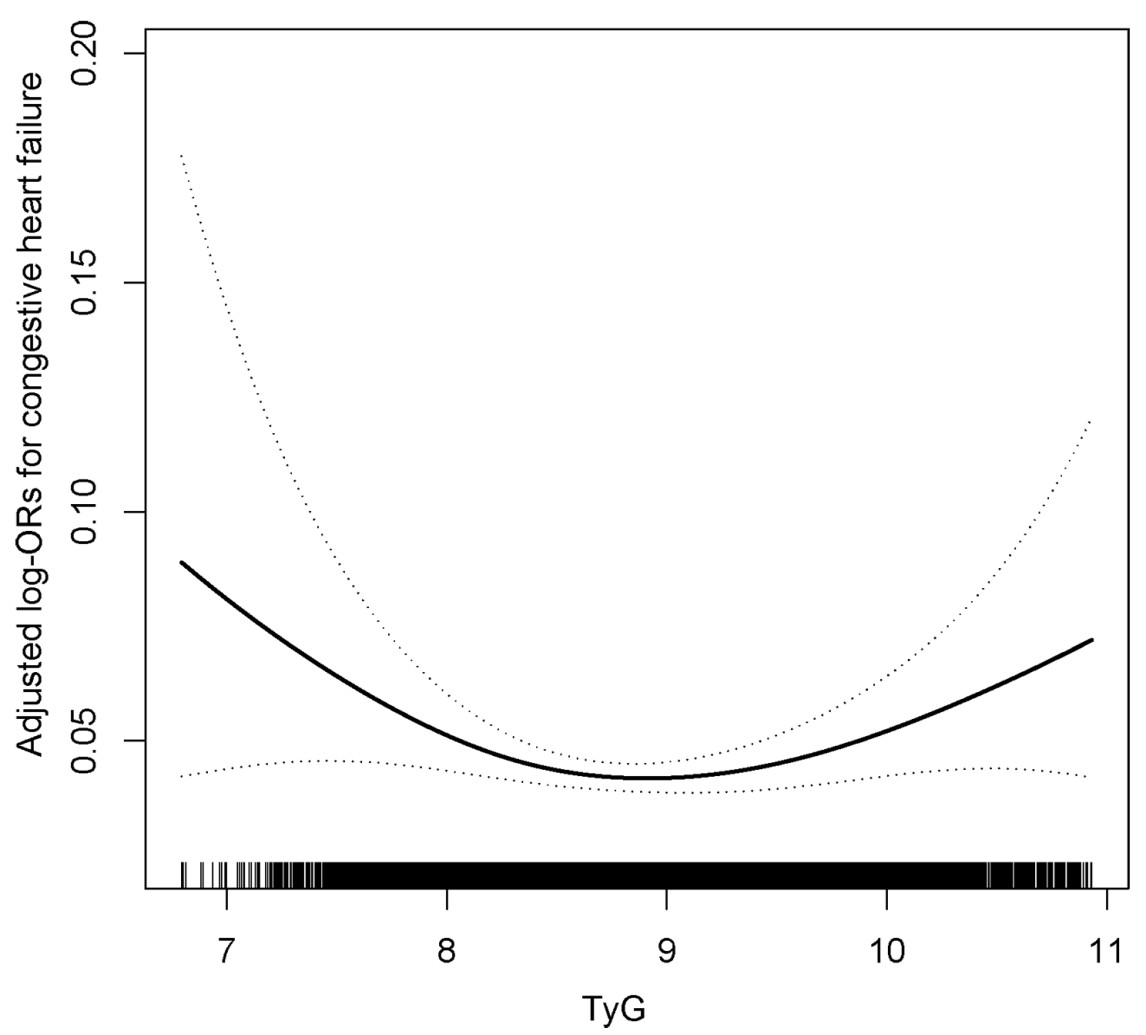
Association between TyG and the prevalence of CHF. A nonlinear association between TyG and the prevalence of CHF was found (*P* < 0.05). The solid line and dashed line represent the estimated values and their corresponding 95% confidence interval. Adjustment factors included age, sex, race, poverty income ratio, BMI, current smoking, HbA1c, eGFR, serum albumin, hypertension, Hyperlipidemia, antihypertensive drugs, lipoprotein-lowering drugs

The multivariate logistic regression analysis in Table 2 demonstrates the association between TyG and CHF. In fully adjusted model 2, each additional unit of TyG is associated with a non-significant odds ratio (OR) of 1.03 (95%CI: 0.88–1.22, *P* = 0.697) for prevelance of CHF. We further stratified TyG into categorized variables. In comparison to the participants in the Q1 group (TyG < 8.40), the ORs for CHF prevalence in the Q2 (8.40 < TyG < 8.81), Q3 (8.81 < TyG < 9.25), and Q4 (TyG ≥ 9.25) groups were 0.82 (95% CI: 0.62–1.09), 0.78 (95% CI: 0.59–1.04), and 0.98 (95% CI: 0.73–1.04), respectively. The trend *P* test > 0.05 suggests the presence of a potential nonlinear correlation between TyG and CHF. Figure 2 visually demonstrates a U-shaped association between TyG and CHF, providing direct evidence for this association. Hence, the utilization of a piecewise logistic regression model is employed to assess the association between TyG and CHF. Two-piecewise logistic-regression model was used to calculate the threshold effect of the TyG (Table 3). If the log likelihood ratio test < 0.05, it means the two-piecewise logistic regression model is superior to the single-line logistic regression model. We observed that the TyG tangent point was 8.60, and on the left side of this point, there existed a negative correlation between TyG and CHF (OR:0.54, 95%CI:0.36–0.81). Conversely, On the right side of the inflection point, we observed a significant 28% increase in the prevalence of CHF per unit increment in TyG (OR:1.28, 95%CI:1.04–1.56 ).

**Table 2:** ORs and 95% CI of CHF incidence according to TyG levels

TyG	Events (%)	CHF OR (95%CI), *P* value^1^	
		Model 1	Model 2
Per 1 unit increment	656 (4.81%)	1.12 (0.96, 1.30) 0.159	1.03 (0.88, 1.22) 0.697
Quartile			
Quartile 1 (< 8.40)	132 (3.87%)	Reference	Reference
Quartile 2 (8.40to < 8.81)	144 (4.22%)	0.87 (0.66, 1.14) 0.304	0.82 (0.62, 1.09) 0.173
Quartile 3(8.81 to < 9.25)	163 (4.78%)	0.87 (0.66, 1.13) 0.295	0.78 (0.59, 1.04) 0.090
Quartile 4 (≥ 9.25)	217 (6.36%)	1.13 (0.86, 1.49) 0.382	0.98 (0.73, 1.31) 0.884
*P* for trend		0.347	0.950

^1^Values are ORs (95% CIs) unless otherwise indicated. TyG, triglyceride glucose index, CHF, Congestive heart failure

Model 1 was adjusted for age, sex, race, poverty income ratio, BMI, HbA1c

Model 2 was adjusted for age, sex, race, poverty income ratio, BMI, current smoking, HbA1c, eGFR, serum albumin, hypertension, Hyperlipidemia, antihypertensive drugs, lipoprotein-lowering drugs

**Table 3:** Results of two-piecewise logistic-regression model

TyG	CHF, OR (95%CI), *P* value^*^
Inflection point (K)	8.60
< K Effect size OR (95% CI)	0.54 (0.36, 0.81) 0.003
≥K Effect size OR (95% CI)	1.28 (1.04, 1.56) 0.018
*P* for log likelihood ratio test	0.001

Two-piecewise logistic-regression model was used to calculate the threshold effect of the TyG. If the log likelihood ratio test < 0.05, it means the two-piecewise logistic regression model is superior to the single-line logistic regression model

^*^Model was adjusted for age, sex, race, poverty income ratio, BMI, current smoking, HbA1c, eGFR, serum albumin, hypertension, Hyperlipidemia, antihypertensive drugs, lipoprotein-lowering drugs

### Subgroup analyses

**Fig. 3:**
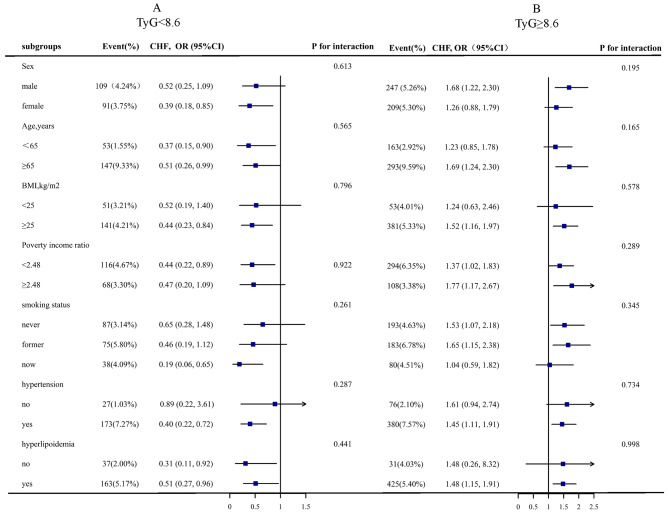
Stratified Analyses by Potential Modifiers of the Association between TyG and the prevalence of CHF * **(A)** TyG < 8.6 **(B)** TyG ≥ 8.6. *Each subgroup analysis adjusted for age, sex, race, poverty income ratio, BMI, current smoking, HbA1c, eGFR, serum albumin, hypertension, Hyperlipidemia, antihypertensive drugs, lipoprotein-lowering drugs. except for the stratifying variable

In order to validate the robustness of our findings, we conducted subgroup analysis and interaction testing (Fig. 3). Considering the U-shaped association between TyG and CHF, we initially stratified the study population into two groups based on the inflection point of TyG at 8.60, followed by a comprehensive evaluation of the continuous relationship between TyG and CHF within different subgroups. The subgroups examined in this study encompassed sex (male vs. female), age(< 65 vs. ≥65), BMI (< 25 vs. ≥25), poverty income ratio (< 2.48 vs. ≥2.48), smoking status (never vs. former vs. now), hypertension (no vs. yes), and hyperlipidemia (no vs.yes); however, none of these subgroups exhibited any significant interaction with TyG (*P for interaction > 0.05* ).

## Discussion

In this comprehensive cross-sectional analysis, we have identified, for the first time, a nonlinear relationship between the TyG index and CHF. Specifically, our findings indicate that among patients with impaired glucose tolerance, both excessively low and high TyG index values are associated with an increased prevalence of CHF. This relationship has been found to be consistent across various subgroups.

The majority of prior population-based investigations examining the relationship between the TyG index and HF have indicated that individuals with the highest TyG index values are at an elevated risk of developing HF [14, 15, 17, 27–29]. Khalaji et al [29]. conducted a comprehensive systematic evaluation on the association between TyG index and HF events, encompassing 772,809 participants from 30 longitudinal cohort studies. The findings revealed a significant positive correlation between elevated TyG levels and increased prevalence of HF in both diabetic patients and those with coronary heart disease. Given that HF represents the advanced stage of numerous cardiovascular diseases, several clinics have demonstrated that TyG, an independent predictor of cardiovascular diseases such as coronary heart disease, stroke, myocardial infarction, and atrial fibrillation, can effectively monitor the progression and prognosis of these conditions [30–33].The aforementioned research enhances the impact of the TyG index on occurrences of HFevents.

The subsequent report presents a comprehensive analysis of the association between TyG and occurrences of HF events. Huang et al. reported that the group with the highest quartile of the TyG index (TyG Q4, with a mean value of 9.5) exhibited a significantly higher risk of developing heart failure compared to the group with the lowest quartile of the TyG index (TyG Q1, with a mean value of 8.0) [14]. Signifcantly, two independent reports also confrmed these results. Xu and coworkers demonstrated that in a population-based cohort of 138,620 participants, Q4 of TyG (9.00-11.65) was signifcantly associated with a higher HF incidence compared with Q1 (6.77–8.16) (*P* < 0.05). Analogously, Zeng et al. reported that in a total of 4992 participants enrolled in the Coronary Artery Risk Development in Young Adults (CARDIA) investigation [from 1985 to 1986 (year 0)], only those participants in the Q4 of TyG (8.3–8.7) were at an increased risk of HF events than those in the Q1 (7.1–7.4) throughout the clinical monitoring timeframe (log-rank test, *P* < 0.001). Remarkably, findings from two separate studies have provided validation for the association between the TyG index and HF incidence.

Xu et al. [15]. conducted a population-based cohort study encompassing 138,620 individuals, revealing that participants within the highest quartile of the TyG index (Q4; range: 9.00-11.65) exhibited a significantly elevated risk of heart failure compared to those in the lowest quartile (Q1; range: 6.77–8.16), with a *P*-value less than 0.05. In a parallel vein, Zeng and colleagues [27], through their analysis of 4,992 participants from the Coronary Artery Risk Development in Young Adults (CARDIA) study, initiated between 1985 and 1986, identified that individuals in the highest TyG quartile (Q4; range: 8.3–8.7) faced a heightened risk of heart failure events relative to those in the lowest quartile (Q1; range: 7.1–7.4) over the course of clinical follow-up, as evidenced by a log-rank test with a P-value less than 0.001. A study conducted in a Chinese population cohort revealed that HF events gradually increased in accordance with the escalation of the TyG index, whether it was considered as a continuous variable or a categorical variable [17]. Conversely, a study in an American population cohort demonstrated that there was a statistically significant difference in the increase of HF events when TyG was treated as a continuous variable. However, when TyG was classified as a categorical variable, the risk of HF events in the Q4 group (TyG ≥ 9) significantly surpassed that of the Q1-Q3 group (TyG < 9) [28] .

However, it is noteworthy that some studies have also demonstrated a lack of association between the TyG index and the incidence of HF [34–36]. Muhammad et al. conducted their study utilizing the Malmö Diet and Cancer Study-Cardiovascular Cohort (MDCS-CV) and The Malmö Preventive Project (MPP) cohorts. They discovered that the increase in the TyG index, when adjusted for potential confounding factors, did not significantly elevate the risk of HF events [35]. Si et al. [36]and their colleagues reported that the TyG index did not exhibit a significant association with HF events in the general population across Europe. Likewise, Jung et al. [34]found that there was no discernible difference in the risk of HF events among cancer survivors, as indicated by a *p* value greater than 0.05.

Our study represents the first to establish a U-shaped nonlinear correlation between the TyG index and HF in patients with diabetes and pre-diabetes. This finding extends beyond the observation that a higher TyG level significantly increases the prevalence of HF. It also provides an explanation for the phenomenon that TyG and HF have been reported to be unrelated in certain research populations. However, it is noteworthy that the nonlinear correlation between TyG and HF was not further evaluated in our study.The observed non-linear correlation between the TyG index and HF may be attributable to the heterogeneity of diseases across different research populations. Consequently, the TyG index may represent the variability in the body’s response to insulin resistance among diverse study groups. In patients with abnormal glucose metabolism, a lower TyG level may elicit a sudden and intense physiological reaction. However, as the TyG level gradually increases, the body may initiate a compensatory process, resulting in a relatively low prevalence of HF. Nonetheless, with a continuous escalation of the TyG level, the ability to compensate for IR may become progressively compromised. To elucidate the specific mechanism underlying the deleterious effect of low TyG levels on myocardial cells in the context of abnormal glucose metabolism, further basic research investigations are warranted.

The development of heart failure associated with high TyG levels may be attributed to the following mechanisms: Hyperglycemia and elevated triglyceride levels can lead to dysregulation of cardiac energy metabolism, thereby increasing the workload on the heart and promoting the progression of HF [37]. Furthermore, insulin resistance can augment the levels of inflammatory mediators, such as tumor necrosis factor-α (TNF-α) and interleukin-6 (IL-6), which may exacerbate inflammatory processes within cardiac tissues and impair heart function [38]. Additionally, IR can induce structural alterations in the heart, including myocardial fibrosis [39, 40].

### Advantages and limitations

The TyG index offers several advantages over the traditional gold standard HOMA-IR for detecting insulin resistance markers in population cohorts, including its simplicity of measurement, repeatability, high sensitivity, and cost-effectiveness. Several limitations of our study warrant acknowledgment. Firstly, due to the inherent nature of cross-sectional studies, we cannot establish a causal relationship between the TyG index and HF. Secondly, although we adjusted for relevant covariates, the possibility of unmeasured confounding factors cannot be entirely excluded. Finally, our research population comprised a representative sample of patients with abnormal glucose metabolism in the United States; therefore, the generalizability of our findings to other populations may be limited.

### Future directions

The findings of this study serve as a cautionary signal for patients with abnormal glucose metabolism. Both excessively high and excessively low levels of the triglyceride-glucose (TyG) index warrant attention, as our results suggest that the TyG index may exert biological effects beyond its association with IR. However, the underlying mechanisms require further elucidation through basic research investigations and large-scale randomized controlled trials (RCTs).

## Conclusions

It is imperative to note that the observed U-shaped correlation between the TyG index and HF prevalence highlights the importance of maintaining optimal levels of this index in patients with dysregulated glucose metabolism. Deviations from the optimal range, either towards higher or lower values, may confer an increased prevalence of HF.

## Data Availability

Publicly available datasets were analyzed in this study. This data can be found here: https://www.cdc.gov/nchs/nhanes/index.htm.
